# Multi-Faceted Environmental Analysis to Improve the Quality of Anthropogenic Water Reservoirs (Paprocany Reservoir Case Study)

**DOI:** 10.3390/s20092626

**Published:** 2020-05-04

**Authors:** Damian Absalon, Magdalena Matysik, Andrzej Woźnica, Bartosz Łozowski, Wanda Jarosz, Rafał Ulańczyk, Agnieszka Babczyńska, Andrzej Pasierbiński

**Affiliations:** 1 Silesian Water Centre of University of Silesia in Katowice, Faculty of Natural Sciences, University of Silesia in Katowice, 40-007 Katowice, Poland; magdalena.matysik@us.edu.pl (M.M.); andrzej.woznica@us.edu.pl (A.W.); agnieszka.babczynska@us.edu.pl (A.B.); andrzej.pasierbinski@us.edu.pl (A.P.); 2 Institute for Ecology of Industrial Areas, 40-844 Katowice, Poland; w.jarosz@ietu.pl; 3 Institute of Environmental Protection—National Research Institute, 00-548 Warszawa, Poland; rafal.ulanczyk@ios.edu.pl

**Keywords:** 3D modeling, global warming effects, algal blooming, chlorophyll a, water management, water quality

## Abstract

Maintaining good condition of dam reservoirs in urban areas seems increasingly important due to their valuable role in mitigating the effects of global warming. The aim of this study is to analyze possibilities to improve water quality and ecosystem condition of the Paprocany dam reservoir (highly urbanized area of southern Poland) using current data of the water parameters, historical sources, and DPSIR (Driver–Pressure–State–Impact–Response) and 3D modeling concerning human activity and the global warming effects. In its history Paprocany reservoir overcame numerous hydrotechnical changes influencing its present functioning. Also, its current state is significantly influenced by saline water from the coal mine (5 g L^−1^ of chlorides and sulphates) and biogenic elements in recreational area (about 70 mg L^−1^ of chlorate and to 1.9 mg L^−1^ Kjeldahl nitrogen) and in sediments (222.66 Mg of Kjeldahl nitrogen, 45.65 Mg of P, and 1.03 Mg of assimilable phosphorus). Concluding, the best solutions to improve the Paprocany reservoir water quality comprise: increasing alimentation with water and shortening the water exchange time, restoration of the 19th century water treatment plant, and wetlands and reed bed area revitalization. The study also proved the applicability of mathematical models in planning of the actions and anticipating their efficiency.

## 1. Introduction

Global climate change causes increasingly severe weather effects throughout Europe. As a result, changes in the environment are observed, which have an increasing impact on citizens’ lives, environment, economy, and agriculture [1,2,3,4]. Such changes can have a great impact on aquatic ecosystems that causes shrinking of water resources (droughts) or, more often, torrential rainfall causing floods or urban flooding. These events result in the reduction of biodiversity by agriculture damage, or loss of fish stock [5]. Residents of large agglomerations also appreciate the role of blue and green infrastructure in mitigating climate change effects. Nature-based solutions use natural capital to provide ecosystem services along with a number of other benefits that address key social, environmental, and economic challenges [6].

A systematic, statistically significant increase in the average annual air temperature is visible in the Upper Silesian Conurbation. It has risen by more than 1 °C over the past 50 years. The number and length of heat waves during which the maximum air temperature exceeds 30 °C also increases. The so-called tropical nights during which the temperature does not fall below 20 °C also occur more often. In the last decades, there has been an increase in the threat caused by several days of heavy rainfall and short-term torrential rains causing floods and, locally, urban flooding [7]. The growing trend for maximum daily rainfall is statistically significant, indicating the possible intensification of heavy rains in the future [8].

Blue-green infrastructure is especially desirable within and around big cities. However, increasing anthropopressure often limits the value of ecosystem services provided by the environments due to the decrease of water quality in the rivers and other reservoirs [9]. Small watercourses and water reservoirs located in urban areas and their immediate vicinity are most at risk. Water reservoirs are often the areas of recreation (fishing, water sports, or walking, running, or cycling routes). This is why the quality of water in this kind of reservoirs and care for their immediate vicinity are of special concern of local authorities. Apart from the recreational function of the reservoirs, there are also other important roles played by the reservoirs, such as water supply for consumption, food production, agriculture, or industry, as well as flood protection. Also, there are other important functions connected with the condition of aquatic systems and water quality such as: maintaining biodiversity and supporting of ecosystems (often of unique character), binding and degradation of pollutants, maintaining appropriate water balance, influencing local microclimatic conditions and mitigating of climate change effects [10,11]. Because of this reason, water reservoirs deserve special attention not only at the local (here, Paprocany reservoir) but also at global scale of the institutions creating environmental protection policy [12,13].

Successful maintenance and protection actions depends inseparably on—and should be preceded by—efficient and constant monitoring. The water quality control has been led to use various methods that depend mainly on the aim and/or economic abilities as well as the time needed to obtain the results. The methods and systems include classical bioindicator studies using sensitive plant or animal species either directly (e.g., [14]) or in regular detection systems or as biosensors [15,16]. More advanced molecular biology techniques are useful for the microbial quality of the water [17,18]. Another group of methods includes the detection of specific chemicals in water, using numerous physicochemical techniques of increasing novelty [19]. Finally, among the most promising monitoring techniques today are those that incorporate nanotechnology, i.e., the use nanoparticles as sensors for pollutants [20,21].

Due to the great significance of the quality of water and water-dependent ecosystems, this issue is the subject of many reports indicating various examples of actions in this area [22]. There are also many methodological tools designed for efficient assessment of water quality [23,24,25,26,27] and the application of this assessment for the systems of aquatic environment management. The assessment of the possibilities to improve the quality of reservoir water resources is an inseparable element of commonly used procedures for water resource management, such as Integrated Lake Basin Management (ILBM) or Drivers, Pressures, State, Impact, and Response (DPSIR)—a method adapted by the European Environment Agency for procedures of water resources management [15,16,28,29]. Increasingly, such assessment is supported by mathematical models that allow for simulation of processes occurring in reservoirs and their catchments. They also help assess potential effects of environmental changes that determine water quality [11,30]. Thus, the models allow for the assessment of the reference state of the environment in order to estimate the influence of analyzed factors (e.g., sources of pollution, duration of water retention) on the water quality. They also enable the scientists to anticipate the efficiency of decisions (catchment and reservoir management) and the consequences of the environmental (e.g., climate) changes on water quality.

The incorporation of biological data into the models (ecosystem models) requires a relatively large amount of data describing the state of the reservoir. These models allow also for the prediction of aquatic organisms’ behavior and interactions under different conditions. Among the most advanced tools there are 3D models (e.g., AEM3D, ELCOM-CAEDYM, GEMSS, GETM) or systems in which it is possible to choose the number of analyzed dimensions according to the purpose of the model application (e.g., Delft3D, EFDC, WASP) [18]. It is currently emphasized that mathematical modeling tools in both hydrology and water ecology should be routinely applied for water resource management and planning [30].

The aim of the work is to indicate actions that may help to improve water quality of anthropogenic urban reservoirs applying extended analyses of the environment of the reservoir and its catchment. As the results of such analyses, proposals for changes in the management of the reservoir and its catchment should be formulated to improve the quality of the water and reservoir functioning. 

As a case study, the Paprocany reservoir (Tychy, Upper Silesia, Poland) was selected, which is exploited and has been subjected to anthropopressure for almost 300 years. In the Paprocany, the DPSIR method was used, including both hydrological and chemical pressures and those arising from the basin management, also in historical terms. Moreover, archival and modern cartographic materials (from 1737 to 2019) as well as hydrological and water quality data collected over the last 25 years were included into the analyses.

## 2. Materials and Methods

### 2.1. Study Area

**Figure 1:**
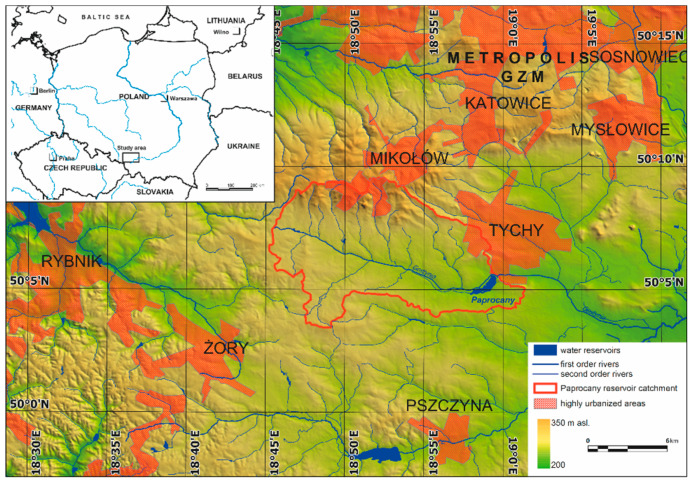
Study area location.

Due to its location, the Paprocany reservoir is an important object that may have an impact on mitigating the effects of climate change in urbanized areas of the southern part of the Metropolitan Association of Upper Silesia and Dąbrowa Basin (GZM). The Paprocany reservoir is located in the city of Tychy in the Gostynia River catchment (left-bank tributary of the Vistula River), in the southern part of Poland, in the central part of the Silesia Voivodeship, which belongs to the most urbanized and industrialized regions in Poland and Europe [31,32,33,34] (Figure 1). Despite structural changes, it is still the area of coal mining and metallurgy industry. Tychy was one of the most dynamically developing Polish cities in the second half of the 20th century—the city’s population in 1990 was over seven times higher than in 1955. Currently, Tychy has almost 128 thousand residents [35].

Paprocany is one of 4773 water reservoirs in the Upper Silesian Anthropogenic Lake District (70.54 water reservoirs per 100 km^2^). In certain parts, up to 200 water bodies are present per 100 km^2^, the average lake density is 2.7% (ratio of water surface area to the total surface area). The water reservoirs either served social and economic functions or were the unintended result of human activity [31,36]. The area of the Paprocany reservoir is 1.051 km^2^ for the normal damming level (242 m a.s.l.), which at present, does not change in time. As a consequence of the constant damming level, the fluctuation of water level, reservoir area and volume of water is not significant (damming volume curve and area curve can be found in “3.1. Cartographic Analysess” chapter). The reservoir was built in the 18th century for the needs of the metallurgical industry to power the water wheels of the “Huta Paprocka” ironworks. The industrial revolution reduced the demand for water and in the second half of the 19th century the reservoir began to perform recreation and fish farming functions that dominate to this day. In addition, the reservoir has a water retention and flood protection function, protecting the south-eastern districts of the city of Tychy. Nowadays, poor water quality and blooms of cyanobacteria in the reservoir significantly limit the development of recreation, water sports, and tourism at the reservoir.

The Gostynia River drains both the areas of industrial (e.g., Łaziska Górne, Tychy) and forest and agricultural areas. The Gostynia catchment is clearly asymmetrical, i.e., left-bank tributaries that flow down from the Silesian Upland predominate. The largest tributary of Gostynia is the Mleczna River, but it flows into Gostynia below the Paprocany reservoir. The hydrographic network of the Paprocany reservoir supply area is less abundant. The main watercourse in this part is Stara Gostynia with small tributaries. Several small watercourses also supply the reservoir directly from the south (Hydrographic Map of Poland, Table 1).

Originally, the supply area of the Paprocany reservoir covered the entire Gostynia drainage basin, closed by the outlet section of the outflow from the reservoir, with an area of 130.6 km^2^. However, its present supply area is limited to 17.94 km^2^, i.e., 13.7% of the original catchment area (see “3.1. Cartographic Analysess” chapter).

While the normal damming level is maintained, the Paprocany reservoir average depth is 1.64 m. Together with a small water supply, it results in a long period of stagnation of water in the reservoir (over 180 days). In summer this leads to rapid heating of reservoir water, which in turn causes a significant acceleration of the appearance of blooms.

Spatial management analysis of the catchment area was performed using Urban Atlas and Corrine Land Cover 2012. Forests, meadows, and anthropogenic infrastructure contribute to the main forms of land use in the reservoir subcatchment. Forests (64% of the area, including: 32%—coniferous, 27%—deciduous forests, 5%—clearcutting and young tree planting areas) are favorable to maintain good water quality in the reservoir because they are natural filters limiting the reservoir’s exposure to the pollution of atmospheric origin. Meadows are located in the valley of the former Gostynia riverbed. Currently, only 26% of the area is utilized (mown or grazed), but not always properly. They should be regularly mown and the swath removed to assure a significant reduction of nutrient runoff into the reservoir. Currently, the cattle grazing and leaving swaths as well as the stable with unprotected leachate of the manure leads to an increased nutrient supply to the Paprocany reservoir.

Housing, road, agriculture and tourist infrastructure, which is potentially a source of nutrients reaching the reservoir with surface runoff, is located in the eastern part of the direct catchment.

Forests that occupy over 48% of the catchment area are the dominant type of use in the upper Gostynia catchment in which the Paprocany reservoir is located. Agricultural and urbanized areas cover about 30% and 10% of the catchment, respectively. Despite the small percentage, industrial areas, and waste dumps and heaps play a key role in determining water quality in the catchment. The basin of the reservoir is occupied mainly by forests, which also dominate in the basin of the Potok Żwakowski Stream and Dopływ spod Chałup Stream.

### 2.2. Data Collection

The protection of the reservoir and its catchment area requires collecting and analyzing the broad scope of data, usually not available at hand. Therefore, there were four main groups of activities executed to achieve the objective: (1) collection and processing of spatial information including historical documents dating back to the 18th century; (2) assessment of the status of surface waters based on the state and local monitoring systems and a monitoring campaign dedicated to this study; (3) detailed characterization of the reservoir using geo referenced sonar sounding and high-resolution water quality probing; and (4) application of the three-dimensional, dynamic model of the reservoir hydrodynamics and water quality in order to assess the impacts of planned measures and climate changes, which may affect the efficiency of measures. The historical and contemporary cartographic sources were shown in Table 1.

To assess the current state of the Paprocany reservoir and its catchment water (1) data from the State Environmental Monitoring from 1995–2014; (2) results of water and sediment testing and monitoring of the quality of the Paprocany reservoir water conducted at the request of the Council City of Tychy in 2004–2006; (3) data of water quality from tributaries of the Gostynia River monitoring; as well as (4) own research were used.

Water quality tests were also carried out at six sensitive points of the Paprocany reservoir and the Gostynia catchment: inflow to the reservoir, pelagic zone, outflow from the reservoir, the mouth of the Potok Żwakowski Stream to the Gostynia, Rów S1 (Ditch S1), which is the saline mine water deposit, and the Gostynia above the mouth of Rów S1 (Ditch S1).

Sonarographic measurements of the reservoir depth were also taken using the bathymetric set, a model of the reservoir bowl and a map of the location and the thickness of bottom sediments developed based on sonarographic measurements of MaxiMapa Co Ltd. the University of Silesia in Katowice [37] using Reffmaster and Surfer 18 software. The purpose of these measurements was to estimate the reservoir capacity at different levels of damming, parameterization of the mathematical model of the reservoir, and to design the measurement network with a multi-parameter probe. 

The analysis of digital terrain model (DTM) of the water catchment area of the Paprocany reservoir was obtained from the Central Geodetic and Cartographic Resource (Main Center for Geodesic and Cartographic Documentation). Merge of DTM images, the water level analysis and terrain profiles and 3D imaging were carried out using the Surfer 18 Golden Software. The analysis of DTM was conducted. In order to assess the spatial variability of the chemical parameters of water of the Paprocany reservoir, a series of measurements of physicochemical parameters was taken using a multi-parameter Hydrolab MS probe that was equipped with sensors for measuring: nitrates, chlorides, dissolved oxygen, water temperature, conductivity, redox potential, and pH. These measurements were carried out at a depth of 0.5 m and in the bottom zone, in a network of squares with a side of 150 m, which allowed obtaining 98 regularly spaced points with known geographical coordinates. Geostatistical methods were used to create the maps of chemical variability [38]. Surfer 18.0 Golden Software was used for data interpolation. Gridding was used with a standard semi-variogram and search neighborhood function.

### 2.3. Model Structure

The Aquatic Ecosystem 3D Model (AEM3D) developed by HydroNumerics was used for the data analysis. The AEM3D is an example of three-dimensional integrated hydrodynamic and ecosystems models. It is based on Estuary, Lake, and Coastal Ocean Model (ELCOM) and Computation Aquatic Ecosystem DYnamics Model (CAEDYM) models developed earlier by the Center for Water Research—the University of Western Australia [39,40,41,42]. The AEM3D allows the users to simulate, among others water flow, water temperature and density, cycles of nitrogen, phosphorus, oxygen, silica, carbon, sediments, metals and organic matter, biomass of bacteria, plankton, and macrophytes [43,44,45].

In this work, the AEM3D model was used to (1) recognize the variability of flow and water quality over time, (2) assess the effects of increased water supply to the tank by transferring water from catchments that do not belong to the current tank supply area, (3) assess the effects of a barrier limiting the water supply to the bathing area in the eastern part of the reservoir, as well as (4) determine the impact of climate change as a factor that may affect the effectiveness of the proposed remedial measures for the reservoir and its catchment. Processes that were included in the model simulations for the Paprocany reservoir are: velocity and directions of the water flow through the reservoir; water retention time (reservoir); transport of a virtual marker, which allows tracking the spread of dissolved substances in water, introduced along with the main tank inflow; water temperature changes; changes in the nutrients and plankton concentration in the reservoir. The output variables that were analyzed in this study include: water retention time, concentration of tracer, and concentration of chlorophyll a.

In the AEM3D model, the transport of mass and energy (hydrodynamics and thermodynamics) are governed by more than a hundred equations and many of them are used to represent chemical and biological processes. Therefore, this chapter describes briefly the main processes included in the model and it is based on the technical documentation [46], while a detailed description of mathematical formulas can be found in the mentioned documentation.

In each time step the model computes the heat exchange in the surface water layer, mixing of scalar concentrations and momentum, wind energy as a momentum source in the wind-mixed layer, the free-surface evolution and the velocity field, horizontal diffusion of momentum, and advection and horizontal diffusion of scalars. The transport equations are the unsteady Reynolds averaged Navier–Stokes and scalar transport equations using the Boussinesq approximation and neglecting the non-hydrostatic pressure terms. The free surface evolution is governed by an evolution equation developed by a vertical integration of the continuity equation applied to the Reynolds-averaged kinematic boundary condition. The scalar (e.g., concentration) transport is based on a conservative third-order method. The heat exchange through the water’s surface is governed by standard bulk transfer models. The energy transfer across the free surface is separated into the nonpenetrative components of long-wave radiation, the sensible heat transfer, and the evaporative heat loss, complemented by penetrative shortwave radiation. The nonpenetrative effects are introduced as sources of temperature in the surface-mixed layer, whereas the penetrative effects are introduced as source terms in water layers on the basis of a decay and an extinction coefficient [46].

Phytoplankton dynamics includes six main processes—such as growth, mortality, respiration, excretion, grazing by zooplankton, and vertical migration. The phytoplankton growth is limited by light, temperature, and availability of C, N, P, and Si. For primary production, the shortwave intensity at the surface is converted to the photosynthetically active component, which is assumed to penetrate according to the Beer–Lambert Law with the light extinction coefficient adjusted to the concentrations of algal, inorganic and detrital particulates, and dissolved organic carbon levels. The C, N, and P cycles, which were mentioned as a limiting factor for the phytoplankton growth are modeled along the degradation of the particulate organic matter to dissolved the organic and dissolved inorganic matter. The nitrogen cycle includes the additional processes of denitrification, nitrification, and fixation. Silica is represented in the model by two forms, i.e., dissolved and algal internal with the phytoplankton uptake and mortality as main driving processes. Zooplankton was also simulated in case of the Paprocany reservoir to balance (limit) the primary production. Main processes simulated for zooplankton are grazing (parameterized primarily by food preferences for phytoplankton, zooplankton, and organic matter), respiration (function of rate coefficient and temperature), losses (mortality, excretion, and egestion), and predation by fish [45].

### 2.4. Specific Model Setting for the Paprocany Reservoir

The model structure is composed of 12 layers of cells with horizontal resolution of 10 m. Seven of these layers are filled with water at the normal damming level. The thickness of layers ranges from 0.47 m in the deepest part to 0.1 m in the layer representing the normal damming level. The model geometry was prepared with the use of above-mentioned sonar sounding data (for the area covered with water at the normal damming level) and 1 by 1-m LIDAR-based digital elevation model (for the area covered with water between normal and maximum damming levels). Meteorological inputs were collected within one year (2016) and included precipitation, wind speed and gust, temperature, pressure, humidity, and cloud cover. The precipitation data were based on six-hour observations, while all of the remaining parameters had a temporal resolution of one hour. The surface inflows to the reservoir were calculated based on the measured flow rate in five cross-sections in streams above the reservoir. The flow rate was measured in a dry season, for which the total flow is considered as a base flow, and in a wet season, for which the rainfall-flow rate functions were calculated. These functions were used to calculate the rain-induced flow based on six-hour precipitation data. There were no sufficient data available for the year of 2016 to parameterize the water quality in the inflows and the initial status of the reservoir. Therefore, based on all of the available water quality observations made in 2016 and before, monthly averaged values were calculated and used as inputs (Table 2).

The simulation covered a period of June–October 2016, which represents a warm season in which the highest concentrations of chlorophyll can be observed. In the following table a set of model configuration parameters is presented (Table 3).

### 2.5. Scenarios Analyzed with the Use of Model

In the case of the Paprocany reservoir, information about the water supply to the reservoir is the most important in terms of water quality and cyanobacterial blooms. It allows conducting variant analysis, in which, apart from the current (real) inflow to the tank, scenarios of partial recreation of the historical reservoir supply area were also analyzed, using the AEM3D model (Table 4).

In all of the scenarios, a constant weir located at the outflow from the reservoir is the section closing the catchment. Water losses resulting from evaporation and connection with groundwater were taken into account. Evaporation was calculated using the AEM3D model based on the evaporative heat flux in accordance to [47] and taking into account the heat transfer coefficient, wind speed, vapor pressure, and water surface temperature [45]. The groundwater contribution was calculated based on the measured difference in the inflow and outflow from the reservoir reported in the “Water management instructions for the Paprocany reservoir” [48], minus the evaporation calculated by the model.

In addition to three scenarios mentioned above, two more (climate scenario 1 and 2) were prepared in order to assess the impact of climate changes on the water retention time, water temperature and concentration of chlorophyll (Table 5). Scenario 0 described above served as a reference (current status) in relation to which the impact of climate changes was assessed. The climate scenarios were based on two “Euro-CORDEX” scenarios, i.e., a moderate climate change scenario—RCP4.5 for the year of 2030 and intense changes scenario—RCP8.5 for the year of 2050. The projections were prepared for the city of Tychy within the project “Development of Urban Adaptation Plans for cities with more than 100,000 inhabitants in Poland” [2,35,36,49]. Input data regarding climate changes included monthly changes in the temperature and precipitation (Table 4). These data were used to modify meteorological inputs to the AEM3D model and to modify rainfall-based inflow rate to the reservoir and air temperature-based temperature of inflows calculated using the appropriate formula [37,50].

### 2.6. DPSIR Methodology

The solutions that lead to the elimination of poor water status require a comprehensive approach to analyzing the causes of such a condition. The method of cause and effect analysis recommended (among others by the European Environment Agency EEA), which comprehensively characterizes the problems, indicates their causes and proposes corrective actions is the DPSIR (Driver–Pressure–State–Impact–Response) analysis [51,52,53,54]. Such analysis consists of five elements: Driving forces of environmental change; Pressures on the environment—environmental burdens generated by activities in the catchment; State of the environment; an effect of pressure on the environment and the economy, including the state of the environment (Impacts on population, economy, ecosystems)—the ecological and economic effect of operations in the catchment and reservoir; Response of the society—actions that are responses to observed phenomena, enabling the maintenance/improvement of the state of the environment and, ultimately, the introduction of appropriate environmental compensations in the studied area.

## 3. Results

### 3.1. Cartographic Analyses

**Figure 2:**
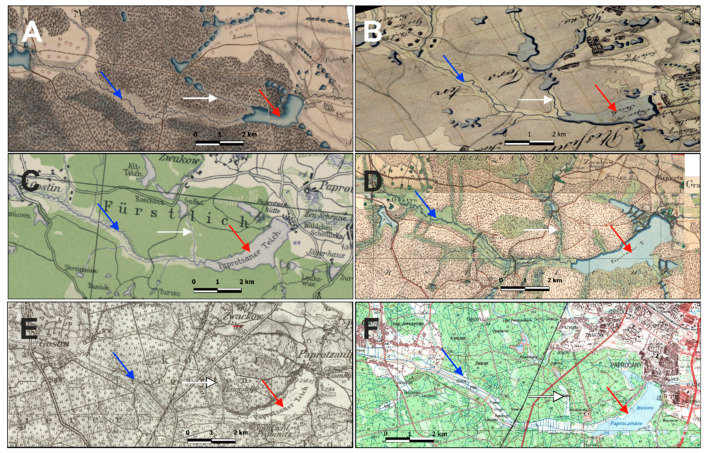
Paprocany reservoir over the centuries—fragments of the map: Christian Friedrich von Wrede 1748 (**A**); Situations Plan von einem Theile Oberschlesiens an der Oestereich – und Neuschlesischen Grenze, Johannes Harnisch, 1794/1795 r. (**B**); Friderizianische Siedlungen rechts der Oder bis 1800 (1933) (**C**); Lieutenant von Sydow from the Border Guard regiment 1827 (**D**); Topographische Karte 1:25,000 (Meßtischblatt 3422 5979) 1944 (**E**); topographic map (**F**). Red arrow shows the Paprocany reservoir, white arrow shows the Potok Żwakowski Stream and blue arrow the Gostynia River.

**Figure 3:**
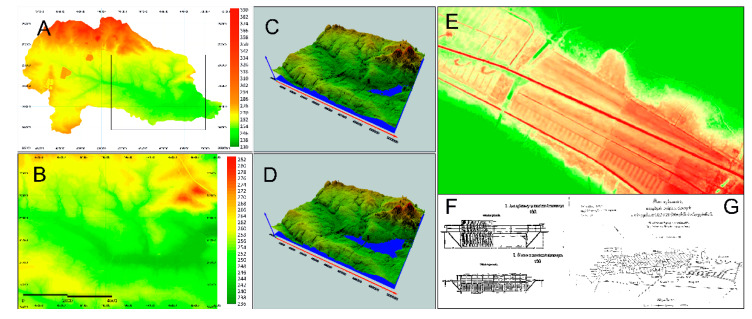
History of the Gostynia River catchment area: Digital Terrain Model of primary catchment area of the Paprocany reservoir (**A**); part of the Paprocany catchment area with the Paprocany reservoir (**B**); 3D model of the catchment area of the Paprocany reservoir at a normal damming level of 242.15 m a.s.l. (**C**) and 3D model of the catchment area of the Paprocany reservoir in the primary damming level of (244.15 m a.s.l.); with visible seepage channels of the root and plant treatment works (**E**); needle weir from 1873, original renovation plan from 1931 (**F**); and original plan of drainage and collection channels from renovation period in 1931 (**G**). 3D image model (**C**,**D**) was carried out on the basis of DTM data using the Surfer 18 Golden Software.

The Paprocany reservoir appeared on the maps of Silesia as early as 1736 (Figure 2A). On the maps made by Schubarth, Mattheus von Wieland in 1736 and by Frederick von Wrede (1748-49) (Figure 2B) the reservoir was larger than today and was fed by the Gostynia River from the west, and by the Potok Żwakowski Stream from the northwest, the waters, which do not end up in the reservoir currently [31]. The analysis of the current digital terrain model (DTM) indicates that the reservoir damming level must have been originally about 2 m higher (Figure 3).

In the 1820s, regulatory works of the Gostynia began, the effects of which can be seen on von Sydow’s maps (1827; Figure 2C). At that time, the reservoir was smaller, and the Potok Żwakowski Stream flowed into the Gostynia, not directly into the reservoir. The Gostynia was divided into two branches: Nowa Gostynia and Stara Gostynia, which partly flowed in the old Gostynia riverbed. The bed of Stara Gostynia has been regulated, and the meadows in the valley have been crossed by a regular network of drainage ditches (irrigation and drainage). The Paprocany reservoir, however, has not been completely cut off from the water resources of this part of the catchment (Figure 2D,E).

**Figure 4:**
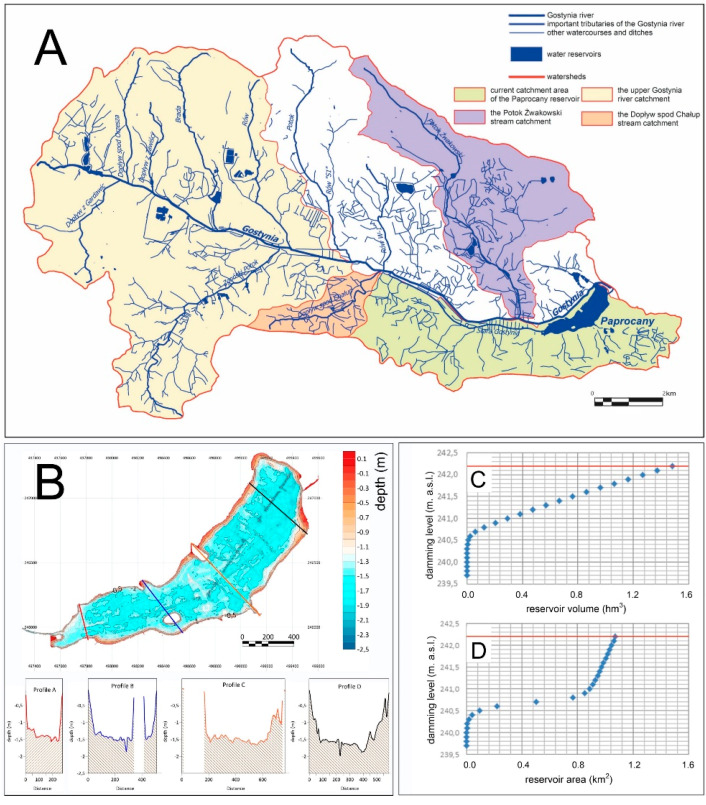
The Gostynia river basin closed with the cross-section of the Paprocany reservoir; the current reservoir catchment and catchment areas were also separated, which were considered for maintenance of (**A**) the reservoir through controlled water metastasis, (**B**) Bathymetric model of fern reservoir with reservoir cross-sections; (**C**) damming curves of the Paprocany reservoir volume; and (**D**) damming curves of the Paprocany reservoir area.

Problems with water quality must have appeared as early as the 19th century. In the 1860s, a soil and plant water treatment system was constructed in the meadows above the reservoir, which purified the waters of the Gostynia (Figure 3F). The wetlands with free water surface are the technology for an effective effluent treatment of aquaculture flow-through systems. Compared with common treatment facilities of flow-through systems, such as microsieves or settling basins, the removal performance of free water surface systems was similar or even higher [38,39]. Thanks to these solutions, for about 120 years, until 1986, the Paprocany reservoir could have been fed, in controlled way, with the waters of the Gostynia River through a channel separated by a weir, connecting New Gostynia (Figure 3) with the reservoir and with the soil and plant treatment works that cleaned the Gostynia waters. According to the designers’ concept, the waters of Nowa Gostynia were introduced to the treatment plant and after filtrating through filtration fields, the water went to, among others, Stara Gostynia and further fed the Paprocany reservoir (Figure 3). From the hydrological point of view, the liquidation of that connection was the most significant change in the method of feeding the reservoir and its water balance, as it caused the complete separation of the majority of the Gostynia River catchment from the reservoir. As the consequence, water inflow to the reservoir got reduced and, in extreme cases, it did not compensate the evaporation. In dry years, the water balance of the tank is negative and there is low, or even interim lack of water exchange rate in the reservoir. The degradation of hydrotechnical devices, and their liquidation, finally caused the separation of the Gostynia catchment into two areas: the south-eastern catchment with an area of about 17.94 km^2^ (Table 6), whose waters feed the Stara Gostynia riverbed and the Paprocany reservoir and the north-west catchment with an area of 83.84 km^2^, supplying Nowa Gostynia, whose waters are currently bypassing the reservoir (Figure 4A).

### 3.2. Impact on the Water Quality

**Figure 5:**
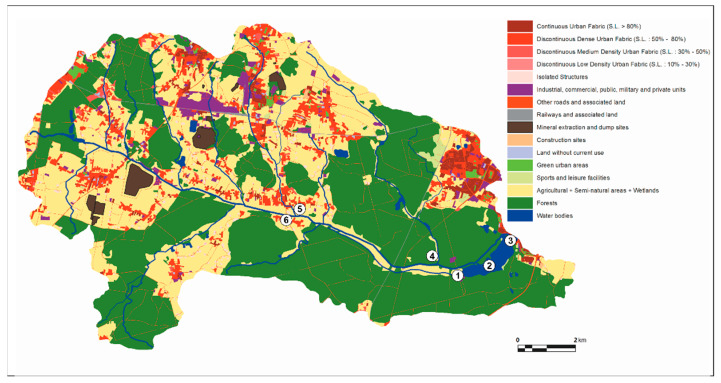
Land cover of the Paprocany reservoir catchment area developed on the Urban Atlas 2012 basis. The number in the white circles shown localization of points of water analysis.

The poor quality of the water of the Gostynia river is clearly demonstrated by the high concentration of sulphates and chlorides, and thus significant values of electrolytic conductivity. These ions can infiltrate the water along the embankment into the reservoir. The source of the salinity is Rów S1 (Ditch S1), where the chlorides and sulphates content periodically exceeded 5 g L^−1^ (Table 7). This is the effect of discharges of saline mine waters from the “Bolesław Śmiały” coal mine, which is located in the northern part of the Gostynia catchment. The high salinity of the Rów S1 (Ditch S1) affects the water in the Gostynia throughout the entire lower section. As a result, the water of the Gostynia on the section from Rów S1 (Ditch S1) to the mouth is not suitable for use as a water source to supply the Paprocany reservoir (Figure 5).

In the central part of the reservoir, there are areas with lower water temperature. This may indicate a natural groundwater inflow in this area of the reservoir. However, the observed changes are negligible.

**Figure 6:**
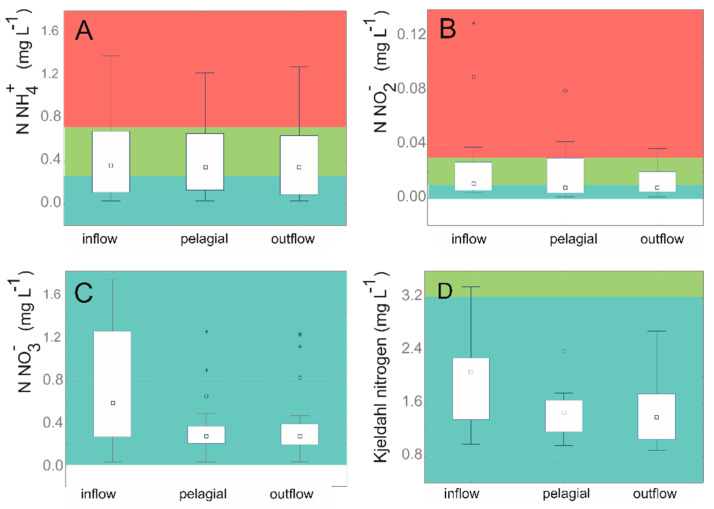
Nitrogen speciation in the Paprocany reservoir water on the inflow, pelagial, and outflow water: (**A**) N-NH_4_^+^; (**B**) N-NO_2_^−^; (**C**) N-NO_3_^−^; (**D**) Kjeldahl nitrogen. Point—median; box—first and third quartile; range—min and max values; asterisk—extreme values. Color in the picture shows the water purity class: blue—class I; green —class II; red—out-of-class water.

In the region of intensive tourist and recreational use, increased levels of nitrates, chlorides, and increased redox potential were observed (Figure 5 and Figure 6). This is probably also related to the inflow located in the south-eastern part of the reservoir draining water from the forest pond. Surface runoff of waters from regions heavily exploited for tourism cannot be excluded. This is indicated by the lack of barriers preventing the entry of nutrients and other substances into the water.

**Figure 7:**
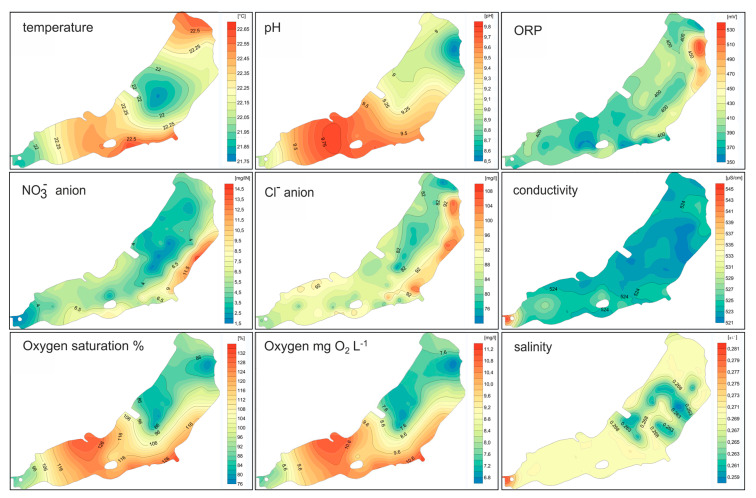
Spatial variability of physicochemical properties of the Paprocany reservoir water.

The analyses showed the isotropic nature of the distribution of the studied phenomena. The analysis of the spatial distribution of temperature (Figure 7) of the water in the reservoir indicates lower temperature in the water inflow zone from the old Gostynia riverbed. This is the area where cold groundwater is pumped into the reservoir and the water comes from the drainage basin along the Stara Gostynia River. This causes a large variation in the measured parameters in this part of the tank. In this region, lower oxygen saturation is also observed, which may indicate a low oxygen content in the groundwater supplied artificially to the reservoir.

In the southwestern part of the reservoir there are regions with an increased pH and dissolved oxygen saturation of water. This indicates a higher photosynthesis activity in this region (Figure 7). Environmental conditions, bioavailability of biogenic elements and temperature in this area stimulate intensive development of algae and cyanobacteria.

The thickness of the sediments of the reservoirs ranges between 4 to 38 cm, and their total estimated volume is about 253 thousand m^3^. This is a relatively small thickness of the sediments. The Paprocany reservoir is characterized by a large bottom area in relation to the volume of the reservoir.

Deposits at the bottom of the reservoir contain a large number of nutrients, the removal of which can significantly improve the quality of the water. It is estimated that the sediments contain 222.66 Mg of Kjeldahl nitrogen (sum of ammonium and organic, non-nitrate nitrogen), 45.65 Mg of phosphorus (P) and 1.03 Mg of assimilable phosphorus in the form of P_2_O_5_. In the situation of proper thermal–oxygen relations, limnic ecosystems accumulate biomass, containing organic matter, as well as nitrogen and phosphorus compounds. The amount of phosphorus and organic matter stored in the bottom sediments is very high. With the loss of the ability to deposit pollution in sediments, the process of so-called internal enrichment begins. It causes a rapid enrichment of water masses with mineral phosphorus compounds, which results in a very rapid increase in primary production (Table 8).

### 3.3. Model-Based Analyses of Mitigation Options

The estimated time of water retention in the reservoir for the current state (scenario 0) is about 55 days in the western part of the tank and about 60 days in the eastern part, including the bathing area and 77 days in its northeastern part. In the case of scenario 1, in which the reservoir is additionally fed with water from the upper Gostynia catchment, the retention time is shortened to 26 days in the western part and 36 days in the eastern part with a maximum value of 58 days in the north-eastern part of the reservoir. In the bathing area, the water retention time is about 40 days. Another increase in the reservoir water inflow, from the Potok Żwakowski Stream (scenario 2), shortens the water retention time to 18, 25, and 47 days, respectively. The retention time in the bathing area is 25 days.

**Figure 8:**
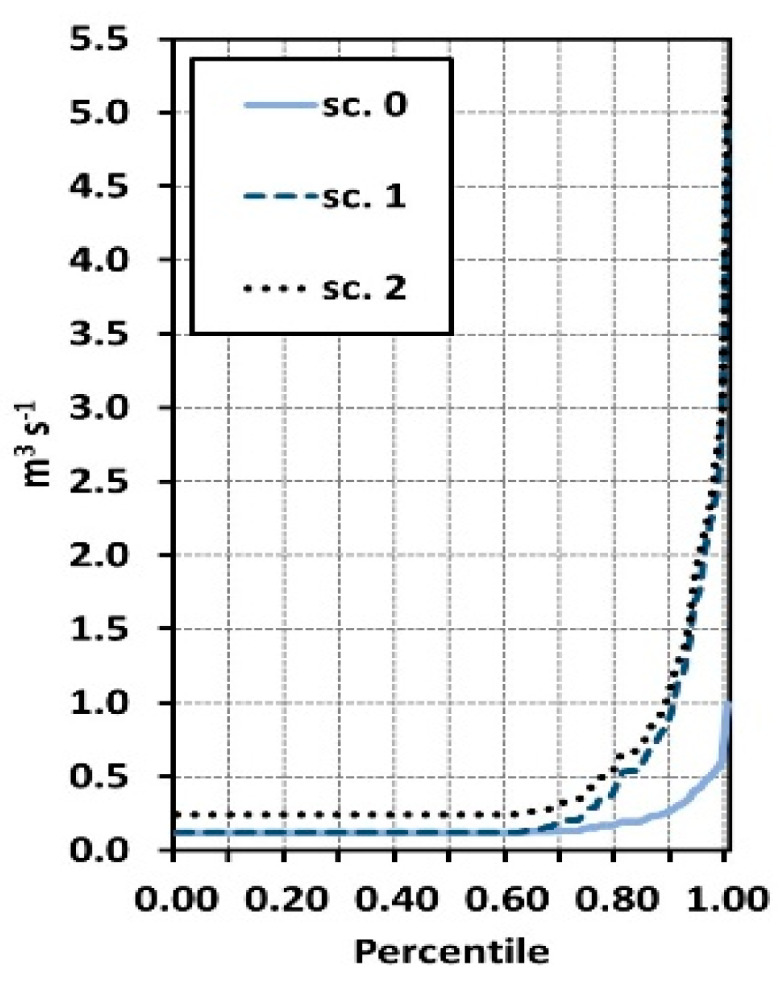
Outflow from the reservoir in scenarios of the restoration of the catchment area (percentile).

Based on the simulations, it can be stated that increasing the tank supply area in accordance with scenario 1 (B) reduces the time of full water exchange in the tank by 25% of the current value, while increasing the supply area following the scenario 2 (C) shortens the water exchange time by 39%. The time of full water exchange is understood here as the time after which the water found in any part of the reservoir flows out. As a consequence of increased inflows, the outflow increases also considerably in analyzed scenarios. The average and maximum rate of outflow increases from 0.167 and 0.994 m^3^ s^−1^ in scenario 0 to 0.374 and 4.887 in scenario 1 and 0.507 and 5.183 in scenario 2. The minimum outflow increases in the scenario 2 in relation to scenarios 0 and 1 from 0.121 to 0.244 m^3^ s^−1^. The percentile of outflows in analyzed scenarios are shown in Figure 8.

**Figure 9:**
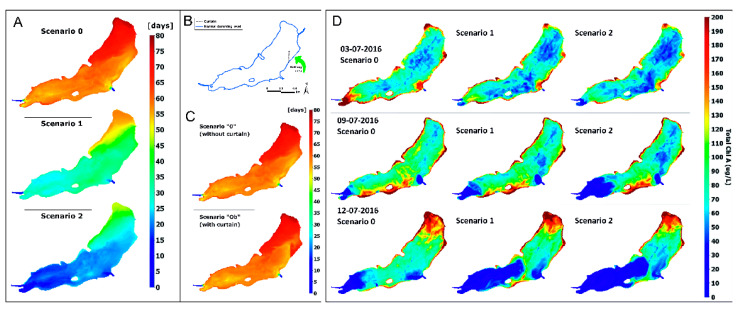
Impact of the catchment area restoration on the water retention time in the Paprocany reservoir (**A**); impact of the curtain on the water retention time (**B**); curtain (barrier) protecting the bathing area from inflow of polluted waters (**C**); chlorophyll concentration change in July according to scenarios 0, 1, and 2 (**D**).

For the three scenarios described above, the impact of the curtain limiting the water flow into the bathing area has also been analyzed (Figure 9). Such curtain was considered as a solution protecting the bathing area from inflow of nutrients and phytoplankton. However, the curtain is supposed to increase the water retention time in the bathing area by four days in the scenario of current inflows (Figure 9). While the longer retention time in the bathing area pose a risk of the water deterioration (warmer water, increased algal growth, decreased oxygen concentrations), the water in northern part of the reservoir will be more intensively mixed due to the installation of the curtain. This positive effect is more significant in combination with the increase in the catchment area (scenarios 1 and 2, Table 5). In scenario 1 with the curtain the maximum water retention time in the reservoir decreases by 25% in comparison to the same scenario without the curtain. In scenario 2, it decreases by 39%.

The impact of restoration of the reservoir’s catchment area (scenarios 1 and 2) on the water mixing is also confirmed by the simulation of virtual tracer which can be considered as a dissolved conservative substance. The tracer was introduced to the reservoir with its surface inflows at the concentration of 1 mg L^−1^. The initial concentration in the reservoir was set to zero. In the current status scenario (Scenario 0) the effect of inflow was barely noticeable in the eastern part of the reservoir in first months of the simulated period. After one month, the concentration in the bathing area was close to zero and after the second month it reached 0.2 mg L^−1^. The average concentration of tracer in the reservoir at the end of five-month simulation was estimated at 0.5 mg L^−1^. In both scenarios which assume partial restoration of the natural catchment area (scenarios 1 and 2) the final average concentration of the tracer ranged from 0.8 to 0.85 mg L^−1^ and was at such level already after 1.5 month.

The simulation of tracer indicated that the curtain may efficiently prevent the inflow of pollutants to the bathing area, however, in a specific condition only. When the quality of inflows is similar to the water quality in the reservoir, the curtain primarily isolates the bathing area increasing the water retention time (especially in the current status scenario). However, when the inflow brings increased concentrations of pollutants (tracer) or increased volume of clean water causing the dissolution of pollutants in the reservoir, the curtain can, respectively, protect the bathing area from pollution until the wind-driven mixing will result in the uniform distribution of pollutant or it will trap pollutants in the bathing area.

To assess the algae bloom risk, the model including inflow scenarios 0, 1, and 2 were also parameterized in a way enabling the simulation of phytoplankton production. This process was simulated taking into account the impact of the nutrients availability, water temperature, light penetration and grazing (according to [39]). The simulation covered the period of June and July 2016, the period in which the increased concentration of chlorophyll a was observed. Calculations allowed to identify locations with the algal blooms. These locations include usually the shallower parts of the reservoir along the banks. Their distribution depends on the inflow rate and, most importantly, on the wind gust and speed. Regardless, the hydrological and meteorological conditions, higher chlorophyll a concentration were present along the water edges in the north-eastern, south-eastern (bathing area), and south-central parts of the reservoir. Lower concentrations were estimated for the deeper parts of the reservoir and for areas close to streams’ inflows—especially in scenarios of the larger catchment area (scenarios 1 and 2) (Figure 9).

**Figure 10:**
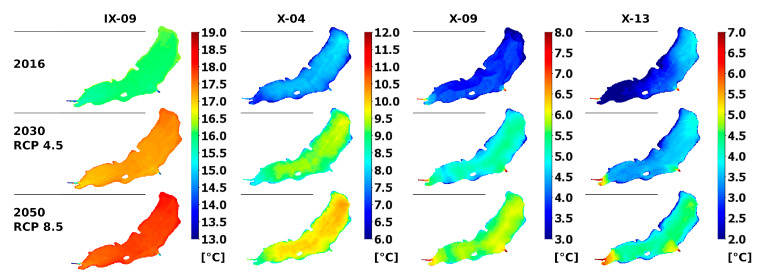
Maps of depth averaged water temperature in September and October 2016 and in two climate change scenarios.

Apart from human activity, also other factors, such as climate change, affecting the efficiency of planned actions should be taken into account. Therefore, two scenarios of change: a moderate (scenario 1) and intense (scenario 2) were analyzed in relation to the current status (scenario 0). In the simulated period (June–October) an average change in the precipitation was estimated at −3.5% and 3.4% for scenarios 1 and 2, respectively. In case of the air temperature, the changes were 0.7 and 1.4 °C. For precipitation, the average inflow to the reservoir in analyzed period was 0.177, 0.175, and 0.179 m^3^ s^−1^ for scenarios 0, 1, and 2, respectively. Similarly, the average outflow from reservoir does not change by more than 1% in relation to the scenario 1, and the maximum outflow is even smaller in scenarios 1 and 2 (by 6 and 3% respectively) due to the decrease in rainfall in scenario 1 and increase in evaporation calculated with the model. Even though the inflow to the reservoir was not expected to change greatly, the temperature of inflows and the heat transfer through the water’s surface affect significantly the water temperature in the reservoir. The average temperature of water in the warm season is expected to increase by 0.6 and 1.3 °C in scenario 1 and 2, respectively. These changes are not uniformly distributed in time and space (Figure 10). The difference of temperature at the outflow ranges from 0 to over 2 °C in scenarios 0 and 2. In various parts of the reservoir, the increase in temperature can exceed 2.5 °C (Figure 10). Expected increase in water temperature due to climate changes can be even greater than presented here, because the simulation did not take into consideration the projected change in the solar radiation.

A temperature rise of one or two degrees should be considered as an important threat making the reservoir more prone to the eutrophication, algal blooms, anoxia, and other adverse effects. It is especially important in the case of shallow reservoirs with considerably high concentrations of nutrients—as the Paprocany reservoir. The risk is even higher because of the climate-driven alteration of the water retention time. The average water retention time is expected to decrease slightly in the scenario 2, however, the beneficial change is observed in model outputs for south-western part of the reservoir only. In the north-east the retention time was estimated to increase.

As a consequence of the factors above-mentioned, the climate change should be seen as a factor limiting the efficiency of corrective measures and posing a risk of much worse ecological status of the reservoir if the corrective measures will not be implemented. It is confirmed by the simulation of phytoplankton cycle in the Paprocany reservoir. The average increase in the chlorophyll a concentration at the outflow was estimated at 7.2% and 3.2% for scenarios 1 and 2, respectively. However, periodically the increase in chlorophyll concentration in parts of reservoir may rise several times (Figure 9).

### 3.4. DPSIR Analysis

It should be noted, however, that the estimated effect of implementing of the recommended methods to improve or maintain water status will be reduced in the longer term due to the predicted climate change. Therefore, the actions should be planned so that they are efficient also in the case of, e.g., increased water temperature, increased evaporation, more intense precipitation and surface runoff, higher (seasonal) plankton production, and other changes resulting from the phenomena above-mentioned. The severity of potential impacts is confirmed by a lot of reviews, and therefore, the water and the catchments management need to take these drivers into account [55,56,57]. These reviews highlight uncertainties related to climate projections and consequently to aquatic ecology modeling, confirming at the same time that the simulation of hydrological and ecological responses to the climate change is the most efficient way to predict upcoming risks and to assess the mitigation measures.

As the main source of the problem, the DPSIR analysis indicates the large trophy of the reservoir and its consequences, however, the causes of this condition are varied and have different sources. The disturbed and unbalanced water relations in the Paprocany catchment should be considered as the main source of problems. They cause that the tank is not supplied with sufficient quality water. The recipe for this condition is undoubtedly the alimentation of water into the reservoir and at least partial restoration of old water relations in the catchment. However, the DPSIR analysis shows that enhancement of the positive effect can also be achieved by proper management in the catchment area (e.g., collecting swaths from meadows, reducing grazing intensity in the immediate vicinity of the reservoir, leachate control) and on the reservoir itself (e.g., sustainable fishing management, care for reed bed in sensitive areas of the reservoir).

The analysis of the causative factors also indicates a potential source of pressure for the reservoir in a situation of increasing its tourist and recreational attractiveness (which will probably take place when the quality of bathing water improves). The influx of tourists will result in increased car traffic and intensified use of recreational and gastronomic infrastructure in the immediate vicinity of the lake. Presently, it is difficult to assess how intense these pressures can be, it should be assumed that the reservoir used this way must be properly monitored in terms of physicochemical parameters of water, and the use of infrastructure in its vicinity should be subjected to a detailed analysis for its adaptation to the growing number of tourists to ensure environmental safety (Table 9).

Solutions that lead to the elimination of poor water status require a comprehensive approach to analyzing the causes of such condition. The method of cause and effect analysis recommended (among others by the European Environment Agency EEA), which comprehensively characterizes the problems, indicates their causes and proposes corrective actions is the DPSIR (Driver–Pressure–State–Impact–Response) analysis. Summary of the research materials, and analyses utilized in this study, and their connection to the DPSIR-framework was presented in the Table 10.

## 4. Discussion

The question of sustainability of urban water reservoirs gains an increasing importance, mainly due to global climate changes and expected shortage of water supply. At the same time, the interdisciplinary studies combining the social, ecological, hydrological aspects that contribute to the social health and cooperation with local authorities connected with water supplies are rare [55].

The main problem limiting the proper functioning of the Paprocany reservoir is the considerably low water inflow resulting from a significant reduction in the supply area which started in the first half of the 19th century. Water supply to the reservoir is one of the basic tools for improving the quality of the water. These types of problems affect many anthropogenic reservoirs in highly urbanized and industrialized areas. Restoration of good water status in many reservoirs will require protection of the catchment area.

As it is stressed generally, the state and monitoring of the catchment area is equally necessary as the possibility of prediction of events and conditions that could change the reservoir status. The applicability of efficient models, which are based on cartographic and GIS-based data for the management of urban reservoirs, where ecological, economic, and social aspect should be met, is exemplified in [19]. The authors conclude that the structure of the land use is reflected then in the hydrographic objects such as reservoirs. The special attention should also be paid to the possibility of using water from sealed areas to supply these tanks. One of the possibilities to improve the water quality of the Paprocany reservoir is to reduce the water retention time and increase the amount of water flowing through the reservoir. Artificial Neural Network (ANN) used for the integration of field data from 10 dam reservoirs in Sri Lanka confirms this kind of attempt [58]. The data analyses demonstrate, as in the present study, the correlation between retention time and chlorophyll a level in the reservoirs. Thus, as the result of dilution obtained in this way a reduction of the concentration of nutrients, mainly phosphorus and elimination of some of the biomass load generated in the reservoir, could be possible. This method is effective for shallow, polymictic, non-stratifying tanks—such as the Paprocany reservoir. To apply this method, it is necessary to deliver good quality water either to the reservoir or to any of the watercourses that flows into it. However, a high level of salinity of the water of the Nowa Gostynia River disqualifies it as a potential source of supply for the reservoir.

The cyanobacterial blooms observed in the tributary area are the consequence of high phosphorous concentration in the Stara Gostynia River. Then, along with the tributary current and with the wind, they are transported along the surface of the reservoir forming a scum containing dead algae and cyanobacteria. The quality of the Gostynia River water is much worse than that of the reservoir, which implied the necessity to search for an additional water supply to the reservoir. Water, potentially acceptable as additional feed of the reservoir, includes the Potok Żwakowski Stream, the Gostynia above the mouth of the Rów S1 (Ditch S1)—Górna Gostynia and the Dopływ spod Chałup Stream. The inclusion of two of these streams to the reservoir’s catchment area was analyzed in the study with the application of the mathematical model, which helped to assess the possibility of decreasing the water retention time and the algal bloom risk. An additional inflow directed to the reservoir could be considered as a factor increasing the flood risk downstream the reservoir. It is worth mentioning, however, due to the limitation of the reservoir catchment, its flood protection function is marginal. Therefore, reduction of the water residence time in the reservoir will not increase the flood risk.

Moreover, a considerable part of surface waters that drain the analyzed area is currently directed to a channel, which transfers the water to the Gostynia River downstream the reservoir. With such existing water transfer, some redirection of water to the reservoir creates a possibility to control the outflow, and thus it reduces the flood risk. Therefore, the restoration of the reservoir’s catchment area will in fact reduce the water retention time, but the total volume of water leaving the analyzed area will not be increased.

Even though the partial restoration of the historical catchment area was assessed to be an efficient measure improving the water quality, for economic and functional reasons, the use of a soil-plant treatment system seems to be the best solution for these purposes [59]. The analyses of historical documents indicate that such solutions have already been used in this area in the past (Figure 2, Figure 3, Figure 4 and Figure 5).

Potentially, restoration of the 19th century historical hydrotechnical devices can be an effective method to improving the quality of the Gostynia River water and it results in feeding the tank with purified water. This would require hydrotechnical operations such as the reconstruction of embankments and damming weirs, carrying out the necessary repairs to the valves, and deepening of ditches that distribute and collect the water from the treatment plant area. It is also required to clean (reconstruct the full section) of the Stara Gostynia riverbed so as to allow water to flow into the reservoir. This can help to improve the quality of the Gostynia water, but also to bring more water to the reservoir. However, the high salinity of the Gostynia water is a big problem. Thus, this solution will be possible only if the discharge of saline mine waters to the Gostynia River tributary is ceased.

The restoration of wetlands would also contribute to the improvement of the quality of Paprocany reservoir water. The water stored in the wetlands is naturally purified as some of the nutrients contained in the water are accumulated in peat. Wetlands and deposits of biogenic sediments are natural reservoirs of organic carbon, which is thus extracted from the atmosphere. Therefore, they have a mitigating effect on the “greenhouse effect” and climate change. However, again—destroyed or neglected wetlands stop storing coal and, themselves, they become a source of greenhouse gas emissions. It is necessary to manage water within the wetlands area in a thoughtful way.

Maintaining the reed bed area is also important for improving the quality of the reservoir water. A reed bed is a biofilter that absorbs phosphorus and nitrogen, biogenic elements for cyanobacteria algae, from the direct catchment of the tank. The reed bed is a natural competitor of cyanobacteria and other photosynthesizing organisms for these elements, reducing the likelihood of blooms. A similar model attempt to restore of a tropic lake in Zimbabwe with field data as the basis for the model, was taken up by [60]. In the model, three scenarios were compared, concerning natural wetlands and the combination of efficient wastewater treatment systems and wetlands as alternatives for the existing management system. According to the model, the combination of the sustainable natural wetland and the wastewater treatment system has the highest potential for the improvement of the water status in the lake. This confirms our recommendation for the restoration of the reed bed area and the water plant system.

The removal of phosphorus by precipitation of iron or aluminum salts, and then depositing of insoluble salts in bottom sediments may be another method to improve the quality of the Paprocany reservoir water. However, the use of chemical agents does not solve the problem in a regular way, but only in the acute incidental situations. A constant supply of nutrients to the reservoir would require a cyclic use of chemical agents. This, in turn, would generate relatively high costs in the long-term perspective.

A curtain considered as an option for the protection of bathing area from algal blooms is estimated to be an efficient solution for a specific hydro-meteorological conditions only. Due to possible periods of increased retention time in the bathing area, installation of such barrier should be supplemented by additional measures, e.g., the regulation of the curtain that is based on the current wind speed and gust and water quality in inflows, aerators, local water mixing, or local water treatment. The installation of the curtain appeared to be efficient in mitigation of algal bloom in drinking water reservoirs in Japan, China, and Korea [61,62,63]. In the Japanese case, the efficiency of control of algal blooms with curtains was also analyzed with the application of mathematical models including DYRESM, which can be considered as a 2D version of the ELCOM model being the predecessor of AEM3D used in the presented study. The methods proposed in [62] seem to be more efficient than tested in the case of Paprocany lake. It suggests that a further analysis of curtain-based remediation methods may be useful, especially with the application of curtains, having depths to cover the epilimnion thickness and curtains covering the entire cross-section of lake.

## 5. Conclusions

In this study, the DPSIR method was used to determine the causes of poor water status and to propose effective remedial actions.

The historical investigation allowed for a reliable assessment of the actions taken up in the catchment area. The analyses included both the pressure generated during the actions and efficiency of the actions.

The large-area analyses of the reservoir with chemical mapping of the water have highlighted the sources of biogenic element contamination of the reservoir (NO_3_)^−^ and places of intensive development of cyanobacteria and algae. This demonstrated that the tools are very useful for the assessment of the causes of the poor condition of reservoir and preparing proposals for adequate remedial actions. In turn, the proposed methods of mathematical modeling of aquatic ecosystems allow to formulate forecasts of the effectiveness of the recommended actions.

For economic and functional reasons, the best solutions to improve the water quality of the Paprocany reservoir are: increasing alimentation with water and shortening the water exchange time, restoration of the 19th century soil and plant treatment system, revitalization of the wetlands, and maintaining and increasing reed bed area.

## Figures and Tables

**Table 1:** Cartographic data sources—historical and contemporary maps and digital data.

Date of Development	Type of Information	Remarks
**Topographic maps**		
1747–1753	Map No. 33 Tychy; Imielin; Mizerów; Oświęcim	Christian Friedrich von Wrede, scale 1:33,333 Krieges-Carte von Schlesien
1747–1753	Map No. 34 Palowice; Żwaków; Pawłowice; Jankowice	
1782	Mapa Hammer 1782	Hand-drawn map of the catchment
1794–1795	Situations Plan von einem Theile Oberschlesiens an der Oestereich und Neuschlesischen Grenze	Johannes Harnisch (copy of Fischer, 1801); Scale—1:120,000
1800/1933	Map Furstenthums Ratibor Pleisner Creifes	Hand-drawn map of the catchment
1806	Massenbach map 1806	Hand-drawn map of the catchment
1827	Kobier blat map 1827	No scale, Lieutenant von Sydow from the Border Guard regiment
1856	Staff map of Pszczyna	Scale 1:100,000 Halemba; Szczakowa; Kobielice; Oświęcim
1881/1883	Staff map of Kobier	Scale 1:25,000 Paprocany Gostyń, Radostowice Jankowice
1906	Zone 5 Kol XX Myslovitz und Oświęcim	Map of Silesia including: Mysłowice, Oświęcim, Mikołów, Bieruń
1933	Polish staff map	Orzesze, Tychy, Gostyń, Paprocany, Scale 1:25,000
1944	German staff map	Scale 1:25,000 Zgoń, Paprocka ironworks, Kobielice, Jankowice
1995	Topographic Map of Poland	Scale: 1:10,000; 1:50,000
**Documentation and plans**		
1872	Repository of ducal files 1870—AKP XI 49, Katowice State Archives, department of Pszczyna	Sketches and technical drawings regarding the development of the catchment, designs of hydrotechnical devices in the Gostynia catchment
1895	Übersichtskarte des Tichauer Baches mit seinem Niederschlagsgebiet im Kreis Pless	Hydrological documentation and river regulation plans
1933	Repository of ducal files 1889–1933—Katowice State Archives, Department of Pszczyna	Documentation of the renovation of hydrotechnical equipment of the catchment
**Thematic map**		
2015	Hydrographic Map of Poland	Scale: 1:50,000 sheet: Tychy, Katowice, Chorzów, Oświęcim
**Digital data and metadata**		
2012	Corine Land Cover (CLC2012)	
2012	Urban Atlas (LCLU 2012)	
2015	Digital Elevation Model	Scale: 1:5000
2018	Hydrographic division of Poland (MPHP)	Scale: 1:10,000

**Table 2:** Input data regarding the inflow and initial conditions in the Paprocany reservoir (units are mg L^−1^ unless otherwise specified).

Parameters		Initial Conditions	Inflow for Months (Monthly Averaged Observations Available for the Reservoir)				
			6	7	8	9	10
Temperature (°C)		18.53	17.867	22.167	21.167	11.917	8.300
Dissolved oxygen		8.59	8.083	7.397	6.700	6.760	6.980
pH		7.66	7.155	7.265	7.240	7.245	7.400
Total suspended solids		9.6	13.375	8.000	29.000	10.050	3.300
Dissolved org. C		6.81	6.217 *				
Particulate org. C		0.88	1.613 *				
Dissolved inorg. C		11.20	11.023 *				
Dissolved org. N		0.89	0.815	0.905	0.880	0.805	0.800
Particulate org. N		0.36	0.36	0.38	0.46	0.53	0.37
Ammonia N		0.29	0.260	0.195	0.680	0.405	0.440
Nitrate N		0.21	0.360	0.200	0.530	0.330	0.820
Dissolved org. P		0.14	0.164	0.123	0.106	0.168	0.130
Particulate org. P		0.06	0.088	0.063	0.079	0.067	0.053
Phosphate P		0.080	0.082	0.076	0.035	0.033	0.054
Silica		1.28	1.368 *				
Bacteria		0.02	0.047 *				
Phyto-plankton (µg Chl a L^−1^)	Mixotrophs	0.25	1.337	2.418	2.649	2.881	2.762
	Cyanobacteria	0.14	0.616	1.115	1.221	1.328	1.273
	Green algae	0.26	2.216	4.006	4.390	4.774	4.577
	Diatoms	0.39	3.656	6.609	7.242	7.876	7.550
Zooplankton (mg C L^−1^)	Predators	0.06	0.071	0.129	0.142	0.154	0.148
	Filtrators	0.07	0.072	0.131	0.143	0.156	0.149

* No data available for the calculation of monthly averaged values.

**Table 3:** Configuration of the AEM3D model for the Paprocany reservoir.

Parameters		Values		
Time step (s)		120		
Mean albedo of the water for shortwave radiation		0.08		
Mean albedo of the water for long wave radiation		0.03		
Wind drag coefficient		0.0013		
Drag coefficient on bottom cells		0.005		
Sediments reflectivity		0.9		
Surface heat transfer coefficient		0.0015		
Light extinction coefficients (m^−1^)	Photosynthetically active radiation	Near infrared	Ultra violet A	Ultra violet B
	1.0	0.2	1.8	2.5
Phytoplankton	mixotrophs	Cyano-bacteria	green algae	Diatoms
Variable internal N and P store	Yes			
Vertical migration and settling type	Motile			Constant
Constant settling velocity (m s^−1^)	-	-	-	−0.12 × 10^−6^
Type of light limitation algorithm	photo-inhibition	no photoinhibition		
Half saturation constant for density increase (uEm^−2^ s^−1^)	-	278	25	-
Rate coefficient for density increase (kgm^−3^ min^−1^)	-	0.9		-
Minimum rate of density decreases with time (kgm^−3^ min^−1^)	-	0.041		-
Rate for light dependent migration velocity (m h^−1^)	0.6	0.3	0.3	0.85
Rate for nutrient dependent migration velocity (m h^−1^)	0.27	0.30	0.30	0.65
Maximum N fixation rate(mg N mg Chl a 24 h^−1^)	0	2	0	0
C:Chlorophyll a ratio	40			
Light saturation for maximum production (µEm^−2^ s^−1^)	390	500	300	100
Initial slope of photosynthesis-irradiance curve (µE m^−2^ s^−1^)	140	150	100	80
Maximum potential growth rate (d^−1^)	1.3	1.0	1.5	3.2
Optimum temperature for growth (°C)	20	20	24	18
Maximum temperature for growth (°C)	28	35	30	30
Standard temperature for growth (°C)	20	20	17	15
Half saturation constant for P	0.001			
Half saturation constant for N	0.05	0.04	0.05	0.04
Maximum internal N concentration (mg N mg Chl*a*^−1^)	12.5	5.0		
Minimum internal N concentration (mg N mg Chl*a*^−1^)	3.5	2.5	3.0	2.7
Maximum internal P concentration (mg P mg Chl*a*^−1^)	0.76	1.50	1.00	0.64
Minimum internal P concentration (mg P mg Chl*a*^−1^)	0.34	0.10	0.30	0.62
Specific attenuation coefficient (µg chl*a* L^−1^m^−1^)	0.02	0.02	0.04	0.04
Minimum density (kg m^−3^)				
Temperature multiplier for respiration	1.04	1.03	1.08	1.07
Respiration rate coefficient (d^−1^)	0.2			

**Table 4:** Summary of scenarios used in the AEM3D model.

Scenario/Description	Inflows to the Reservoir	Catchment Area (km^2^)	Inflow to the Reservoir in the Simulated Period (m^3^ s^−1^)		
			Average	Minimum	Maximum
S0 Current drainage area (reduced in relation to the natural one)	Main inflow	8.45	0.075	0.055	0.423
	3 small southern streams	9.39	0.102	0.076	0.581
	Scenario 0	17.84	0.177	0.131	1.004
S1 Is the scenario 0 and additional transfer of water excess from the Rów S1 (Ditch S1) above the minimum flow (baseflow 0.216 m^3^ s^−1^)	Upper Gostynia River	61.18	0.207	0	3.893
	Scenario 1	77.84	0.384	0.131	4.897
S2 the scenario 1 and additional transfer of water from the Potok Żwakowski stream	Potok Żwakowski stream	18.83	0.134	0.123	0.483
	Scenario 2	96.67	0.517	0.254	5.193
Climate scenario 1	Scenario 0	17.84	0.175	0.131	0.946
Climate scenario 2	Scenario 0	17.84	0.179	0.131	0.977
Map of catchment areas for scenarios	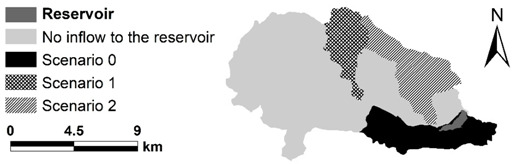				

**Table 5:** Climate change scenarios analyzed for the Paprocany reservoir.

Climate Scenario	1	2	1	2
Euro-CORDEX Source Scenario	RCP4.5	RCP8.5	RCP4.5	RCP8.5
Year	2030	2050	2030	2050
Month	Change in the Average Monthly Air Temperature (°C)		Change in the Monthly Sum of Precipitation (%)	
1	0.8	1.6	−8.9	−7.1
2	0.8	2.2	13.7	36.1
3	0.4	1.4	−9.0	−5.4
4	−1.1	−0.1	67.6	84.3
5	−0.5	0.2	−12.2	2.9
6	0.0	0.7	−10.2	−10.0
7	0.2	0.9	−6.6	−3.1
8	0.6	1.4	−15.7	−3.3
9	1.4	1.8	5.0	24.0
10	1.5	2.3	35.1	49.4
11	0.0	0.8	−0.6	−2.5
12	0.5	1.0	56.2	53.8
Average	0.4	1.2	9.5	18.3

**Table 6:** Basic parameters of catchments computational.

Catchment Name	Catchment Area (A in km^2^)	Average Annual Precipitation (P in mm)	Average Annual Unit Runoff (SSq in dm^3^ s^−1^ km^−2^)	Average Annual Runoff (SSQ in m^3^ s^−1^)
Current catchment area of the Paprocany reservoir	17.94	730	7.2	0.130
Upper Gostynia River	61.18	758	8.3	0.510
Potok Żwakowski Stream	18.83	750	8.8	0.167
Dopływ spod Chałup Stream	3.83	740	8.5	0.032

**Table 7:** Classification of water quality in the Paprocany reservoir and the catchment of the Gostynia River, markings of water purity class: blue—class I; green—class II; red—out-of-class water (analyses based on the data provided by the City of Tychy, the Regional Inspectorate of Environmental Protection in Katowice and by authors’ analyses).

Indicator	Unit	Inflow	Pelagial	Outflow	Potok Żwakowski Stream Mouth to Gostynia	Rów S1 (Ditch S1)	Gostynia River above the Mouth of the Ditch S1
No. according to Figure 5		1	2	3	4	5	6
Nitrogen N-NH_4_	mg L^−1^	0.135	0.108	0.110	0.526	0.401	0.557
Nitrogen N-NO_3_	mg L^−1^	<0.01	<0.01	<0.01	0.042	0.310	0.070
Nitrogen N-NO_2_	mg L^−1^	0.210	0.193	0.021	2.090	5.820	1.590
Nitrogen Kjeldahl’s	mg L^−1^	0.92	1.19	1.05	3.22	6.59	2.73
Chlorate	mg L^−1^	79.4	70.9	65.9	29.8	4750.7	97.9
Phosphate P-PO_4_	mg L^−1^	<0.01	<0.01	0.021	0.022	0.315	0.030
Magnesium Mg	mg L^−1^	7.18	6.86	6.69	10.63	89.58	12.23
pH		6.97	7.83	7.54	8.16	8.23	7.83
Conductivity	µS cm^−1^	468	430	432	484	12261	1162
Sulphate SO_4_	mg L^−1^	-	-	72.6	322.1	337.5	308.6
Total Dissolved Solids	mg L^−1^	-	-	304.4	330.0	8252.0	622.0
Calcium Ca	mg L^−1^	40.14	37.77	37.36	66.34	146.00	85.56
Total Organic Carbon	mg L^−1^	7.83	9.22	9.06	6.63	3.69	5.34

**Table 8:** Estimated amount of sediments and nutrients accumulated in them in the Paprocany reservoir (own calculations).

	Unit	Value
Total sediment volume	(m^3^)	253,028.0
Sediments surface	(m^2^)	1,550,911.0
Sediments thickness	(m)	0.23
Sediments wet weight	(Mg)	27,833.08
Kjeldahl Nitrogen	(Mg)	222.66
Fosfor (P) in the sediment	(Mg)	45.65
Fosfor (P_2_O_5_) in the sediment	(Mg)	1.03

**Table 9:** DPSIR analysis of the Paprocany reservoir.

Driver	Pressure	State	Impact	Response
Climate changes	Temperature rise; Increased duration of drought period in the catchment area; Change in the nature of precipitation—extreme rainfall events	Increase of water temperature in the reservoir; Increasing retention time and reducing inflows in dry periods due to the change in the precipitation characteristics and increased evaporation; Increased load of sediments and pollutants due to the more intensive rainfall events and runoff	Induction of phytoplankton blooms, and biomass accumulation in the sediments; Periodic increase in nutrient concentration in the water; The ecosystem services value decreased	Increased supply of good quality water to the reservoir
Historical factors	Limiting the surface of the tank catchment area causes too low water inflow to the reservoir	Water stagnates in the reservoir, the supply of nutrients after precipitation and evaporation causes an increase in the concentration of nutrients	Increased nutrient content in the reservoir resulting in phytoplankton blooms	Feeding good quality water into the reservoir, increasing the reservoir basin, restoring (at least partially) the former water relations in the basin
Agricultural activity in the direct catchment of the Paprocany reservoir	Inflow of waters enriched with nutrients and surface runoff from agricultural areas, especially after rainfall, leaving swaths in meadows	Increased nutrients levels: N–NH_4_; P–PO_4_	Enrichment of sediments in the reservoir, induction of phytoplankton blooms	Mowing meadows and removing hay bales, preventing the decomposition of matter, limiting intensive grazing in the direct catchment of the tank, controlling leachate in the catchment
Mining industry (hard coal mines)	High salinity of waters that could be used to supply water to the reservoir	High saline waters	Inability to use heavily saline waters to feed water to the reservoir	The need to use watercourses that are not fed with mine waters
Fishing on the Paprocany reservoir	Restocking with calm-prey fish, restocking with adult predatory fish	Incorrect age structure of calm-prey and predatory fish populations, ecological imbalance	Lack of natural relations between the environment of the reservoir and fish, disturbed circulation of matter	Restocking in accordance with the fisherman’s survey, educational activities directed at fishing environments
Limiting of the rushes area	Limiting of the rushes area due to the development of recreational infrastructure, concrete hardening of quays, acceleration of water/biogens surface runoff	Increased nutrients levels: N– NH_4_; P–PO_4_, TOC	Enrichment of sediments in the reservoir, induction of phytoplankton blooms	Recreation of rushes on the banks of tanks, preferring the creation of unpaved/water-permeable areas around the tank
Intensification of recreational use of the Paprocany reservoir	Increasing the intensity of functioning of centers providing services related to recreation and catering outlets, increasing car traffic and thus deposition	Potential possibility of increasing the inflow of nutrients, detergents, deposition from car and petroleum exhaust gases	Increasing pollution of the lake’s waters with various substances with different environmental impacts	Conducting ongoing control of the use of the area around the Paprocany reservoir and water monitoring, responding to potential and existing pressures affecting reservoir waters

**Table 10:** Summary of the research materials, and analyses utilized in this study, and their connection to the DPSIR-framework.

	Materials	Methods/Analyses	Modified DPSIR
History	Table 1	Screening of archive materials	Driving forces; Pressures; State; Impact; Responses; Goals
Water quality (Paprocany reservoir and Gostynia basin)	Data from Long term water quality monitoring of water quality (WIOŚ, City of Tychy)	Analysis of existing data sets analyzing reports	State; Impact
Limnology of the Paprocany reservoir	Limnological and Hydrobiological Studies of the “Paprocany” Water Reservoir 2005 report		Driving forces; Pressures; State; Impact; Responses; Goals
Land cover	Digital Terrain Model Landsat TM, satellite images were used for spatial development analyses	Spatial analyses Analyses of infrastructure development	Pressures; State; Impact; Responses; Goals
Official water management documents			Pressures; State; Impact; Responses; Goals
Spatial analysis of water quality of the Paprocany reservoir	Short-term, spatial investigations of water conditions series of measurements of water physicochemical parameters	Field analysis Spatial statistical analyses	Pressures, State
Sediment		Sonographic analysis of sludge thickness, qualitative and quantitative chemical analysis	State; Impact
Bathymetry		Sonarographic bathymetry analysis	State
Fisheries	Fishing and stocking data (2013–2015)	Own research (8 and 9 November 2016), electro fishing technology	State
Ground water	Data on the quantity and hydrochemical parameters of ground water	Not available	
Stream flow	Periodic measurements of the flow rate in tributaries in various meteorological conditions	Preparation of rainfall-runoff relation for tributaries	Impact; State; Responses
Futures studies	Scenarios regarding climate change (EuroCORDEX RCP scenarios) and restoration of the catchment area	GIS-based estimation of possible water transfer scenarios; model-based simulations of the water flow, water temperature, retention time and water quality	Driving forces; Pressures; Impact; State; Responses; Goals
